# Salvianolic Acid A Ameliorates Early-Stage Atherosclerosis Development by Inhibiting NLRP3 Inflammasome Activation in Zucker Diabetic Fatty Rats

**DOI:** 10.3390/molecules25051089

**Published:** 2020-02-28

**Authors:** Quanxin Ma, Qinqin Yang, Jiaojiao Chen, Chen Yu, Lizong Zhang, Weimin Zhou, Minli Chen

**Affiliations:** 1 Institute of Comparative Medicine & Experimental Animal Research Center, Zhejiang Chinese Medical University, Hangzhou 310005, China; 2 Department of Experimental Animals, Zhejiang Institute of Traditional Chinese Medicine, Hangzhou 310007, China

**Keywords:** atherosclerosis, salvianolic acid A, Zucker diabetic fatty rat, NLRP3 inflammasome

## Abstract

Salvianolic acid A (SAA), an important bioactive polyphenolic acid found in *Salvia miltiorrhiza* Bunge, may be used for treating metabolic disorders due to its anti-inflammatory activity. Since chronic inflammation plays an important role in type 2 diabetes mellitus (T2DM) complicated with atherosclerosis (AS), SAA may have beneficial effects on AS. Here, we evaluated the effects of SAA on metabolic disorders in male Zucker diabetic fatty (ZDF) rats induced by a high-fat diet and Vitamin D3 injections. Compared with the model group, the SAA high dosage (1 mg/kg) group exhibited decreased hemoglobin A1C levels but unchanged blood glucose levels. The disrupted lipid profiles were ameliorated by SAA, with significantly decreased levels of blood cholesterol, LDL-C and triglyceride. The protective effects of SAA against early AS were further confirmed by histopathological examination of aortic tissues. In addition, we observed that SAA decreased serum hs-CRP levels and suppressed the activation of NLRP3 inflammasome and NF-κB signaling in aortic tissues of ZDF rats. Collectively, our results demonstrate the potential of SAA to alleviate AS and T2DM in ZDF rats as a result of its anti-inflammatory effects.

## 1. Introduction

Diabetes mellitus is a common endocrine metabolic disease that affects millions of adults worldwide. The number of diabetes patients has nearly quadrupled over the past thirty years, from 108 million patients in 1980 to 422 million patients in 2014, and this number is estimated to reach 592 million patients in 2035 [1]. Among them, type 2 diabetes mellitus (T2DM) accounts for the majority of patients(> 85%) [2]. Diabetes can cause a variety of serious complications, including blindness, renal failure, cardiovascular disease, and peripheral gangrene. Macrovascular diseases are common complications in T2DM. Atherosclerosis (AS), one of the most devastating cardiovascular diseases, is the major cause of disability and death in T2DM patients [3]. Nevertheless, the current medical treatment methods to reduce the morbidity and mortality of T2DM with AS have limitations. Therefore, research studies of the pathogenesis and treatment strategies for diabetic AS are urgently needed.

In recent years, a large number of studies have shown that in addition to lipid metabolism disorders, inflammation is the common pathological basis for T2DM and AS [4,5,6]. Chronic inflammatory response runs through the entire development process of AS in T2DM. In the early stage of the disease, endothelial cells are stimulated to produce E-selectin, P-selectin and vascular cell adhesion molecule-1 (VCAM-1) under the combined effects of high insulin and oxidized low-density lipoprotein (ox-LDL) levels [7]. Subsequently, monocytes are recruited by the cytokines, penetrate deep into the subvascular endothelium area, are activated into macrophages, and phagocytose lipids to form foam cells. After the formation of foam cells, the cells are enriched under the endothelium, while inflammatory cytokines are released. The final rupture releases lipids, and forms atherosclerotic plaques [8]. Therefore, controlling inflammation in T2DM patients may be beneficial for inhibiting AS in T2DM.

*Salvia miltiorrhiza* Bunge is a traditional Chinese medicine that is often used for the prevention and treatment of cardiovascular diseases. Salvianolic acid A (SAA) is an important, water-soluble phenolic compound found in *Salvia miltiorrhiza* Bunge [9]. In recent years, a number of preclinical studies have found that SAA has a certain preventive effect on diabetes and its related complications [10,11,12]. From the literature review and our own research results, we found that SAA has significant anti-inflammatory activities. This anti-inflammatory activity may manifest in regulating a number of different pathways, such as inhibiting the activation of the NF-KB signaling pathway, inactivating the P2 × 7r-Pkr-Nlrp3 signaling pathway, and regulating the composition of the intestinal flora [13,14,15]. In addition, SAA can improve the dysfunction of vascular endothelial cells and the remodeling of vascular structures [16]. We therefore hypothesize that SAA has therapeutic potential to prevent AS in T2DM. In this study, we established an animal model of T2DM with early AS by a high-fat diet plus intraperitoneal injections of VD_3_ to evaluate the effects of SAA on early Zucker diabetic fatty rats. 

## 2. Results

### 2.1. Effects of SAA on the Body Weight and Food Intake in T2DM ZDF Rats with AS

**Figure 1:**
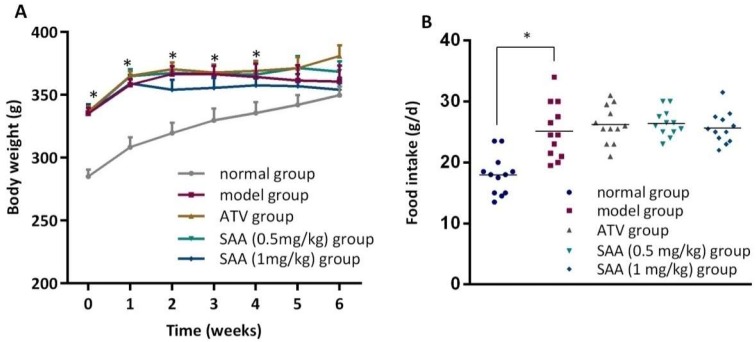
ZDF rats were fed with experimental high-fat diets (HFD) for 4 weeks, and were then injected intraperitoneally with 6 × 10^5^ IU/kg of VD_3_ (three times, every three days). The drugs were administered to the ZDF rats daily starting after the first VD_3_ injection for 6 weeks. (**A**) shows the effects of SAA on body weight changes; (**B**) shows the effects of SAA on average daily food intake. SAA, salvianolic acid A; ATV, atorvastatin. Data are expressed as mean ± SEM, n = 6. * *p* < 0.05 vs. normal group (Zucker lean control rats).

The body weight represents the animal’s basic status. As shown in Figure 1, the body weight of the ZDF model group was higher than that of the normal group from pre-administration (at week 0) to the 4th week of administration (*p* < 0.05). However, there was no significant difference in body weight between the ZDF model group and the ZL control group at week 5 and week 6 (*p* > 0.05). In addition, we did not see any significant differences in body weight between each drug-treated group and the model group (*p* > 0.05). Because food intake is closely related to the blood glucose and blood lipid levels, we observed food intake for two days in the 6th week of administration. The results showed that food intake in the ZDF model group was higher than that in the ZL control group (*p* < 0.05), and no significant differences were seen between the drug-treated groups (*p* > 0.05). Thus, SAA did not have any effects on the body weight and food intake in ZDF rats.

### 2.2. Effects of SAA on Blood Glucose and HbA1c Levels in T2DM ZDF Rats with AS

**Figure 2:**
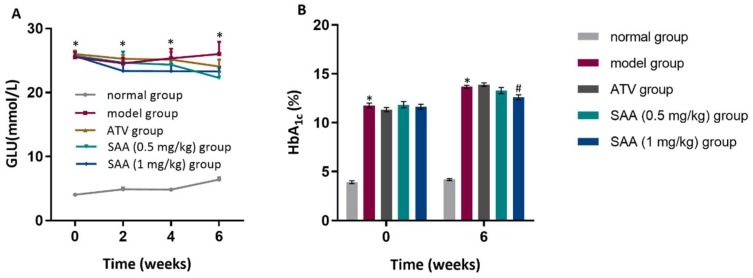
Effects of SAA on blood glucose and HbA1c in T2DM ZDF rats with AS. ZDF rats were fed with experimental high-fat diets (HFD) for 4 weeks, and were then injected intraperitoneally with 6 × 10^5^ IU/kg of VD_3_ (three times, every three days). The drugs were administered to the ZDF rats daily, starting after the first VD_3_ injection for 6 weeks. (**A**) shows the effects of SAA on blood glucose levels; (**B**) shows the effects of SAA on blood HbA1c levels. SAA, salvianolic acid A; ATV, atorvastatin. Data are expressed as mean ± SEM, n = 6. * *p* < 0.05 vs. normal group (Zucker lean control rats); ^#^ *p* < 0.05 vs. model group (Zucker diabetic fatty rats with HFD and VD_3_ injections).

Blood glucose, blood lipids and hemoglobin A1C (HbA1c) levels were higher in the ZDF groups than those in the normal group before administration and after 2 weeks, 4 weeks and 6 weeks of administration (*p* < 0.05), as shown in Figure 2A. The blood glucose levels of the SAA high-dose group and the SAA low-dose group were not significantly different from that of the model group (*p* > 0.05). When compared with the model group (Figure 2B), the SAA high-dose group showed lower HbA1c levels after 6 weeks of administration, while no differences were observed in the SAA low-dose group or in the positive group (*p* > 0.05). Moreover, we found an increase in insulin sensitivity index (HOMA-IR and HOMA-IS) in the ZDF rats when compared with the ZL control rats, but did not see any significant differences in the SAA group or the ATV group. Therefore, it can be inferred that SAA did not improve glucose metabolism or insulin resistance in ZDF rats.

### 2.3. Effects of SAA on Blood Lipid Levels in T2DM ZDF Rats with AS

**Figure 3:**
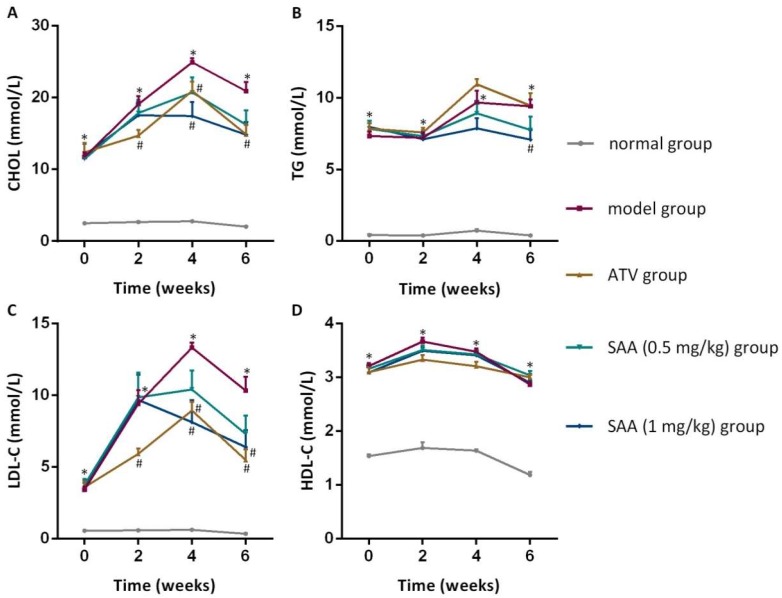
Effects of SAA on blood lipid levels in T2DM ZDF rats with AS. ZDF rats were fed with experimental high-fat diets (HFD) for 4 weeks and were then injected intraperitoneally with 6 × 10^5^ IU/kg of VD_3_ (three times, every three days). The drugs were administered to the ZDF rats daily, starting after the first VD_3_ injection for 6 weeks. The effects of SAA on cholesterol (CHOL) levels (**A**), triglyceride (TG) levels (**B**), LDL-C (low density lipoprotein-cholesterol) levels (**C**) and HDL-C (high density lipoprotein-cholesterol) levels (**D**) are shown. SAA, salvianolic acid A; ATV, atorvastatin. Data are expressed as mean ± SEM, n = 6. * *p* < 0.05 vs. normal group (Zucker lean control rats); ^#^ *p* < 0.05 vs. model group (Zucker diabetic fatty rats with HFD and VD_3_ injections).

As shown in Figure 3, the CHOL, TG, LDL-C and HDL-c levels in the ZDF model group were higher than those in the normal group before administration and after 2 weeks, 4 weeks and 6 weeks of administration (*p* < 0.05). The levels of CHOL and LDL-C in the SAA high-dose group at the 4th and 6th week of administration were significantly lower compared to those in the model group (*p* < 0.05), whereas the level of TG was significantly lower at the 6th week of administration (*p* < 0.05). Furthermore, significant decreases in the Atherosclerosis Index were found in the SAA group and the ATV group (Table 1).

### 2.4. SAA Ameliorated Histopathological Changes in the Aortic Tissues of T2DM ZDF Rats with AS

**Figure 4:**
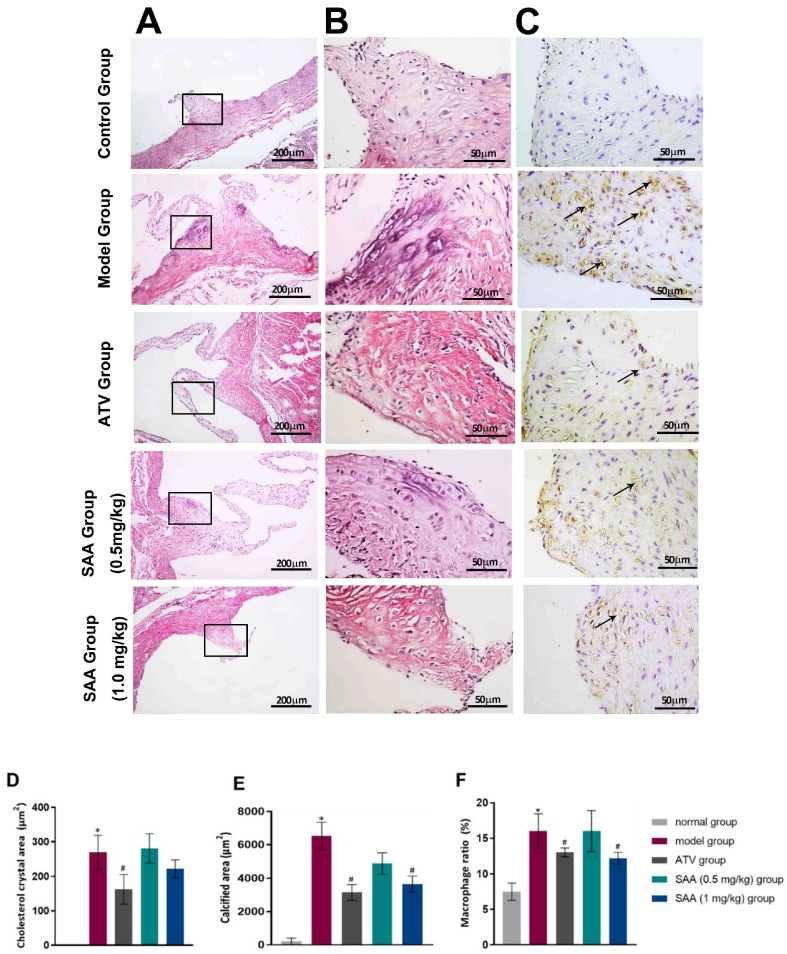
SAA ameliorated histopathological changes in the aortic tissues of T2DM ZDF rats with AS. The ZL and ZDF rats were sacrificed, and the aortic tissues were collected for H&E staining (**A** line, 200×. **B** line, 400×) as well as IHC staining (**C** line, 400×). Representative activated macrophages in the aorta were stained with anti-CD68 and are indicated by black arrows. Histopathological quantitative analysis was performed by experienced pathologists. The data of cholesterol crystal area (**D**), calcified area (**E**) and macrophage ratio (**F**) are shown. Data are expressed as mean ± SEM. * *p* < 0.05 vs. normal group (Zucker lean control rats). ^#^ *p* < 0.05 vs. model group (Zucker diabetic fatty rats with HFD and VD3 injections).

H&E staining showed that the endothelium was smooth in the root of the aortic valves without obvious thickening or inflammatory cell infiltration, and the smooth muscle cells were arranged neatly in the ZL control group. In contrast, the foam cells and the cholesterol crystals aggregated in the root of the aortic valves, and the smooth muscle cells had a disorganized arrangement with partial calcification in the ZDF model rats. Nevertheless, fewer foam cells, cholesterol crystals, and calcification were formed in the SAA low-dose group, the SAA high-dose group, and the ATV group. Macrophage infiltration was not observed in the aortic tissues of the normal rats, but was observed in the aortic tissues of the ZDF model rats (Figure 4A,B). Compared to the model group, the SAA-treated group and the ATV-treated group showed a reduced level of macrophage infiltration in the aortic tissues (Figure 4C).

### 2.5. Effects of SAA on Serum Hs-CRP in T2DM ZDF Rats with AS

The level of serum high-sensitivity C-reactive protein (hs-CRP) content was not significantly different between the normal group and the model group before VD_3_ injection (data not shown). The serum hs-CRP level in the model group significantly increased compared to that in the normal group at week 2, 4 and 6 after the first VD_3_ injection (*p* < 0.05), as shown in Table 2. The hs-CRP level in the SAA low-dose group and the SAA high-dose group was significantly lower than that in the model group and the ATV group after 4 and 6 weeks of administration (*p* < 0.05). However, the hs-CRP level in the positive group was lower than that in the model group and the ATV group at the 2nd and 4th week of administration (*p* < 0.05). 

### 2.6. Effects of SAA on the Expression of NLRP3 and Caspase-1 in the Aorta Tissue of T2DM ZDF Rats with AS

**Figure 5:**
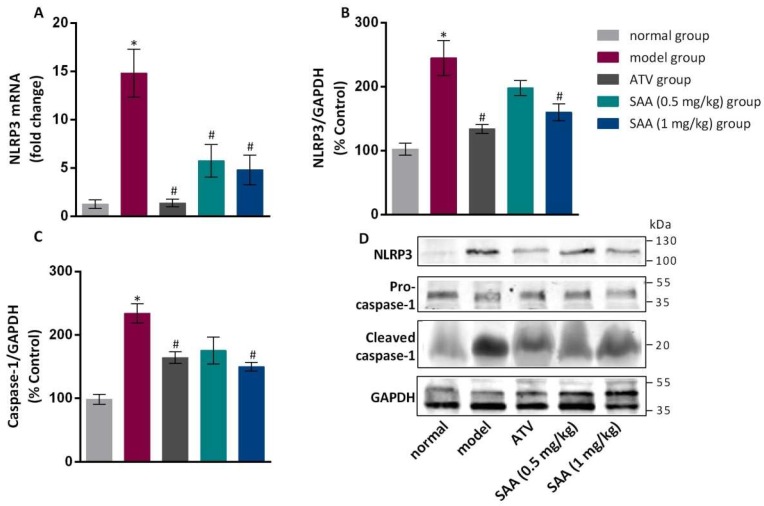
Effects of SAA on the expression levels of NLRP3 and caspase-1 in the aortic tissue of T2DM ZDF rats with AS. ZL and ZDF rats were sacrificed, and the aortic tissues were collected for gene and protein expression analysis. The effects of SAA on NLRP3 mRNA (**A**) and protein (**B**) expression are shown. (**C**) The effects of SAA on caspase-1 mRNA expression are shown. Data are expressed as mean ± SEM (n = 6). * *p* < 0.05 vs. normal group; ^#^ *p* < 0.05 vs. model group. (**D**) Western blotting showed the expression levels of NLRP3 and cleaved caspase-1 protein in each group.

Compared with the normal group, the model group showed significantly increased expression of NLRP3 (both mRNA and protein levels) in the aortas (*p* < 0.05), as shown in Figure 5. The expression level of NLRP3 was significantly lower in the aortas of the positive group and the SAA high-dose group compared to that in the model group (*p* < 0.05). The expression level of the caspase-1 precursor protein was not significantly different between the groups (*p* > 0.05), while cleaved caspase-1 showed differential expression. Specifically, the level of cleaved caspase-1 was significantly higher in the model group compared to that in the normal group. In the SAA high-dose group and in the positive group, the cleaved caspase-1 level ws markedly lower compared to that in the model group (*p* < 0.05).

### 2.7. Effects of SAA on the Expression Levels of NF-κB and IL-1β in the Aortic Tissue of T2DM ZDF Rats with AS

**Figure 6:**
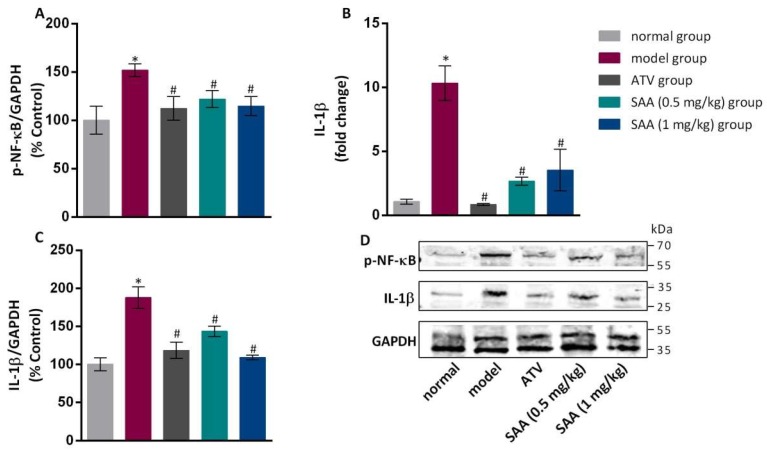
Effects of SAA on the expression levels of NF-κB and IL-1β in the aortic tissues of T2DM ZDF rats with AS. ZL and ZDF rats were sacrificed, and the aortic tissues were collected for gene and protein expression analysis. (**A**) The effects of SAA on p-NF-κB expression are shown. (**B, C**) The effects of SAA on IL-1β mRNA and protein expression are shown. The data are expressed as mean ± SEM (n = 6). * *p* < 0.05 vs. normal group; ^#^ *p* < 0.05 vs. model group. (**D**) Western blotting showed the p-NF-κB and IL-1β protein expression levels in each group.

Compared to the normal group, the model group showed an increased level of phosphorylated NF-κB protein in the aortic tissues, as well as increased levels of IL-1β mRNA and protein (*p* < 0.05), as shown in Figure 6. The expression levels of phosphorylated NF-κB and IL-1β were significantly lower in the aortic tissues of each treated group than those in the model group (*p* < 0.05).

## 3. Materials and Methods

### 3.1. Experimental Animals and Chemical Reagents

Twenty-four male Zucker diabetic fatty (ZDF) rats aged 7–8 weeks and six male Zucker lean (ZL) rats of the same age were purchased from Beijing Vital River Laboratory Animal Technology Co., Ltd. (SCXK, Beijing, 2012-0001, China). All animals were housed in standard conditions at the Zhejiang Chinese Medical University Laboratory Animal Research Center (SYXK, Hangzhou, 2013-184, China). Each individual ventilated cage (IVC) housed two rats with food and water supplied ad libitum. The experiment was conducted after one week of adaptive feeding. The experimental protocols conformed to the guidelines of the Laboratory Animal Research Center of Zhejiang Chinese Medical University (SYXK, Hangzhou, 2008-0116, China) and the Use Committee. ZL rats were fed with a standard AIN-93G diet. ZDF rats were fed with a high-fat diet (1% cholesterol, 10% lard oil, 10% yolk powder, 0.5% No. 3 cholate and 78.5% AIN-93G diet).

SAA powder (purity > 99%) was provided by Chiatai Qingchunbao Pharmaceutical Co., Ltd. (Hangzhou, China). Atorvastatin (ATV) was obtained from Pfizer Pharmaceutical Co., Ltd. (New York, NY, USA). A rat hs-CRP enzyme-linked immunosorbent assay (ELISA) kit was purchased from Chengwei Biological Technology Co., Ltd. (Hangzhou, China). The blood biochemical reagents were purchased from Shanghai Shengneng Desai Diagnosis Technology Co., Ltd. (Shanghai, China). The CD68 antibody (ab31630) was obtained from Abcam Company (Cambridge, MA, USA). The anti-NLRP3 (SC-34408), anti-caspase-1 (SC-56036) and anti-GAPDH (SC-166545) antibodies were obtained from Santa Cruz Biotechnology (Santa Cruz, CA, USA), and the anti-p-NF-κB (#3033) antibody was obtained from Cell Signaling Technology (Danvers, MA, USA).

### 3.2. Establishing a ZDF Rat Model of T2DM with AS 

**Figure 7:**
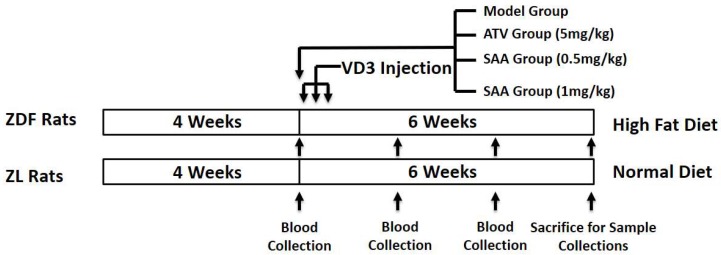
Experimental design. ZDF, Zucker diabetic fatty; ZL, Zucker lean; ATV, atorvastatin; SAA, salvianolic acid A.

Twenty-four 8-week-old male rats were fed with a high-fat diet continuously for 4 weeks. After fasting for 12 h, blood was taken from the submandibular vein for testing the serum levels of glucose (GLU), CHOL (cholesterol), TG (triglyceride), LDL-C (low density lipoprotein-cholesterol), and HDL-C (high density lipoprotein-cholesterol). All ZDF rats were divided into a model group, an atorvastatin (ATV) group (positive control, 5 mg/kg b.w., p.o.), a SAA low-dose group (0.5 mg/kg b.w., tail vein i.v.) and a SAA high-dose group (1 mg/kg b.w., tail vein i.v.), based on the blood glucose, blood lipid and body weights (n = 6 per group). Six male ZL rats of the same age were used as the normal group and received a standard diet. ZDF rats of the model, ATV, SAA low-dose and SAA high-dose groups continued to receive a high-fat diet and were injected intraperitoneally with 6 × 10^5^ IU/kg of VD_3_ (three times, every three days) to induce AS [11]. The drugs were administered after the first VD_3_ injection. The ZL rats from the normal group, the model group and the ATV group were injected with the same amount of saline solution by tail vein injections. The administered drug volume of all groups was 5 mL/kg b.w. The experimental diagram is shown in Figure 7.

### 3.3. Measurement of Blood Biochemistry

For blood biochemistry, 1 mL of blood was taken from the submandibular vein of each rat (after fasting for 12 h) at week 2, 4 and 6 after the first VD_3_ injection. The serum was obtained through centrifugation of the blood sample. GLU, CHOL, TG, LDL-C and HDL-C levels were tested by an auto-analyzer. The atherosclerosis index (AI) was calculated as AI = (CHOL—HDL-C)/HDL-C. The hs-CRP and insulin contents were detected by ELISA kits (Chengwei Biological Technology Co., Ltd., Hangzhou, China; Merck Co., Ltd., Darmstadt, Germany).

### 3.4. Histological Evaluation of the Aortas

All animals were anesthetized and sacrificed at week 6 after the first VD_3_ injection. The heart was taken close to the root of the ascending aorta after being washed with precooled PBS through the left ventricle. Then, the heart was put into a 10% neutral formaldehyde solution for 48 h to fix the tissue. The heart was dissected along the coronal plane under an auricular appendage. The ascending aorta of the heart was embedded from the root section down. The root of the aortic sinus was sliced into 4 μm sections using a microtome. After hematoxylin and eosin (H&E) staining of the slices, the pathological changes were observed and photographed under the Nano Zoomer S60 Digital Pathology System (Hamamatsu Co., Ltd., Shizuoka, Japan). The cholesterol crystal area and calcification area were measured by NDP.View 2.7.39 software (Hamamatsu Co., Ltd., Shizuoka, Japan).

Macrophage infiltration in aortic tissue was tested by immunohistochemical staining. The paraffin sections were heated at 60 °C for 1 h in an oven. After deparaffinization, the endogenous peroxidase was blocked with 3% H_2_O_2_ for 15 min. Then, the sections were incubated with the primary anti-CD68 antibody (abcam, ab31630) overnight at 4 °C in a refrigerator. After washing, the sections were incubated with the secondary antibody for 15 min. Next, a freshly prepared diaminobenzidine (DAB) solution was applied to visualize the antibody, followed by hematoxylin staining, dehydration and mounting. The macrophages were stained brown.

### 3.5. RNA Extraction and Quantitative Real-Time PCR (qPCR)

Frozen vascular tissue (40–60 mg) was taken, from which all RNAs were extracted and synthesized into cDNA by reverse transcription. The primer sequences of NLPR3 were 5′-GCTAAGAAGGACCAGCCAGA-3′ and 5′-CCAGCAAACCTATCCACTCC-3′. The primer sequences of GAPDH were 5′-GCTAAGAAGGACCAGCCAGA-3′ and 5′-CCAGCAAACCTATCCACTCC-3′. Quantitative real-time PCR was performed using a two-step amplification method, and all information was collected by the IQ5 PCR instrument (Bio-Rad, Hercules, CA, USA). As the GAPDH housekeeping gene was used as an internal control, we calculated the transcriptional levels of target genes using the 2^−△△Ct^ method based on cycle threshold (Ct) values.

### 3.6. Western Blotting Analysis

Frozen vascular tissue (50 mg) was taken from the same section, from which proteins were extracted using the KeyGEN Total Protein Extraction Kit (KeyGEN BioTECH, Nanjing, China) from KeyGEN BioTECH (Nanjing, China). The protein content was quantified using the bicinchoninic acid (BCA) method (KeyGEN BioTECH, Nanjing, China). After adding the sample buffer, the protein content was further boiled for 5 min at 95 °C. The boiled protein samples (10 μL) were added to each well, and then sodium dodecyl sulfate-polyacrylamide gel electrophoresis (SDS-PAGE) was performed. Subsequently, the proteins were transferred to nitrocellulose membranes. The membranes were then blocked with 3% albumin from bovine serum albumin (BSA) at room temperature for 2 h, followed by overnight incubation with primary antibodies at 4 °C. After being washed with TBS-T for four times, the membranes were incubated with secondary antibodies at room temperature for 1 h. The nitrocellulose membranes were rinsed three times for 5 min each in a shaker without light, and TBS was used for the last rinse. At the end, the nitrocellulose membranes were scanned using an Odyssey infrared fluorescent scanner (Thermo Fisher, Waltham, MA, USA). The protein bands were analyzed, and the gray values were calculated using Quantity One software (4.6.8, Bio-Rad, Hercules, CA, USA).

### 3.7. Statistical Analysis

Experimental results were analyzed using the SPSS 19.0 statistical software (Armonk, NY, USA), and are expressed as the mean value ± the standard error ($\bar{x}$ ± SEM). Statistical analyses, including one-way analysis of variance (ANOVA) and L-S-D analysis for group comparisons, were performed using Prism 5 (GraphPad Software, San Diego, CA, USA). *p* < 0.05 was considered significant.

## 4. Discussion

The incidence of T2DM has increased rapidly worldwide and has resulted in several vascular complications that harshly impacted the finances and life quality of afflicted individuals. AS is known as the major macrovascular disease that occurs under diabetic conditions [17]. Medicinal plants and their active components have great potential in preventing and treating metabolic disorders, including T2DM and AS. Recent evidence has suggested that SAA relieved diabetes complications [18], such as vascular disease and peripheral neuropathy, though few studies have been performed to support the applications of SAA in the treatment of AS in T2DM patients.

In this study, the early stage of the AS model was established by feeding a high-fat diet to accelerate T2DM pathogenesis in HFD-fed ZDF rats, followed by periodically injecting VD_3_ to facilitate AS formation 4 weeks after [19]. ZDF rats are a well-studied animal model for metabolic disorders studies, such as T2DM. Male ZDF rats that were fed with a HFD developed obesity and insulin resistance within 4 weeks, while hyperglycemia continued to develop with age [20]. Nevertheless, rats are not able to develop vascular abnormalities when fed with only a HFD due to special lipid metabolism. VD_3_ injection helps to promote the formation of AS because it induces a calcium overload in the aorta, which damages arterial endothelial cell integrity and leads to the invasion of inflammatory cells into the bloodstream [21].

Our experimental results showed that serum glucose had a sharp increase and was maintained at a higher level after 4 weeks of a high-fat diet. Nevertheless, SAA did not have a modulating effect on the blood glucose level. This finding disagrees with a previous study which showed that SAA lowered the levels of blood glucose after fasting and after food intake in a T2DM mouse model induced by a high-fat and high-sucrose diet and streptozotocin injections [18]. The discrepancy between our study and this previous study might be attributed to the animal model that was used. HbA1c reflects the average blood glucose level of the past 8 weeks, which can be regarded as a predictor of vascular diseases and mortality in T2DM [22]. We noticed that SAA reduced HbA1c levels after 6 weeks of administration, suggesting that SAA has long-term antidiabetic effects.

Dyslipidemia is a cardinal symptom in T2DM that causes lipoprotein to rise in AS [23]. Disorders of lipid metabolism in patients with AS are mainly manifested by a decrease in HDL-c and an increase in LDL-c. [24]. After penetrating into the endothelium, LDL is modified in a variety of ways and is then ingested by macrophages to form foam cells; this is the mechanism of LDL facilitating AS formation [25]. The foam cells gather gradually, and fatty streaks are formed; this accelerates the migration and proliferation of vascular smooth muscle cells and fibroblasts. Then, LDL penetrates continually into the surface of the fiber cap to form necrotic nuclei, and the plaques are prone to rupture [26]. Hence, lowering the LDL levels has become the main strategy to prevent and treat AS [27]. Our study found that serum lipid levels, especially LDL levels, were decreased significantly by SAA. The TG content was lower than that in the model group after 6 weeks of administration, suggesting that SAA regulated dyslipidemia. This is consistent with the findings of Qiang [28]. Based on a histological analysis, the results demonstrated an aggregation of foam cells and cholesterol crystals, and showed that macrophage infiltration occurred in the roots of the aortic valves of ZDF rats.

The anti-AS effects of SAA may be related to the regulation of HbA1c and blood lipids. Another question is whether SAA can play a prominent anti-inflammatory role in early AS-ZDF rats to intervene in the development of AS. We dynamically observed the serum levels of hs-CRP in each group of rats from 0 week of administration to 6 weeks of administration. Hs-CRP is a key biomarker of inflammation that can reflect and predict the inflammation level of the body and the risk degree of AS [29]. It was found that the serum hs-CRP levels increased significantly in the model group, indicating that the body had a chronic inflammatory response [30]. In our study, we observed markedly decreased hs-CRP levels after 4-6 weeks of SAA administration, which suggests that long-term intervention by SAA reduces the inflammation reaction.

**Figure 8:**
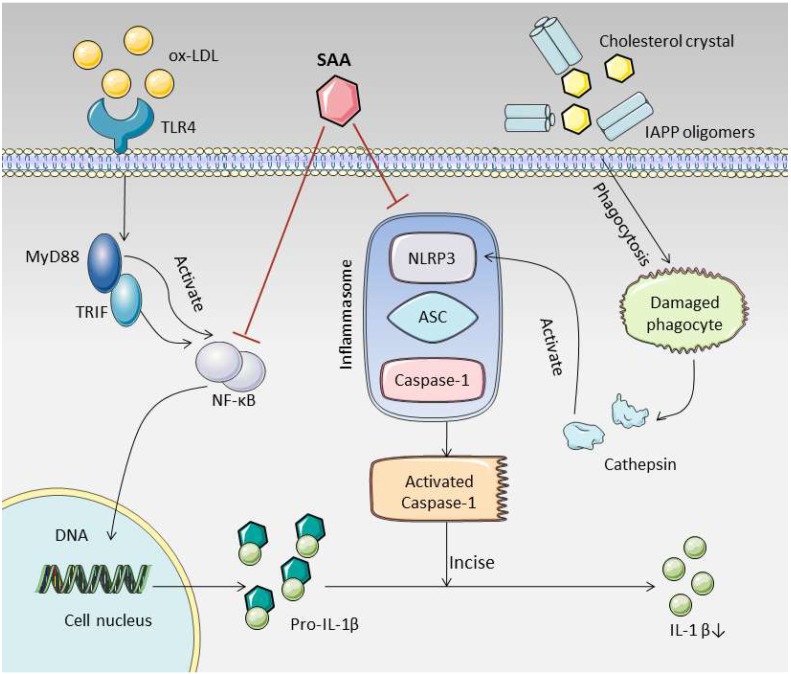
Salvianolic acid A ameliorates early-stage atherosclerosis development by inhibiting NLRP3 inflammasome activation in Zucker diabetic fatty rats. SAA can reduce the activation of NLRP3 inflammasome and NF-κB, thereby reducing the production of cleaved caspase-1 and IL-1β. This may be an important pathway for SAA to combat early AS in T2DM. (The material in the picture comes from https://smart.servier.com/).

The role of NLRP3 inflammasome in AS has attracted increasing attention and has become a potential target for the treatment of AS [31]. Activated NLRP3 inflammasomes in early plaques can lead to a series of inflammatory responses [32]. As the protein expression level of NLRP3 increased, the cholesterol crystals and islet amyloid polypeptide (IAPP) oligomers that were produced in T2DM cases with AS lesions were engulfed by macrophages, which caused the lysosomes to rupture and to release cathepsin. Cathepsin regulates the activation of NLRP3 to form polymers and provides a platform for the activation of caspase-1 [33]. Caspase-1 is an important protein component of the NLRP3 inflammasome complex, which usually exists in the form of an inactive zymogen (pro-Caspase-1). Once activated, it gathers with NLRP3 and ASC to assemble into the NLRP3 inflammasome complex. After that, pro-Caspase-1 is hydrolyzed, and the activated cleaved caspase-1 is formed through a series of reactions [34,35]. We measured the expression levels of pro-caspase-1 and cleaved caspase-1, and found that the level of pro-caspase-1 was not significantly different between the groups. The level of cleaved caspase-1 was found to increase in the model group and to decrease after SAA intervention, indicating that SAA could reduce the production of activated cleaved caspase-1. Phosphorylated NF-κB enters the nucleus and increases the transcription level of IL-1β. Caspase-1, activated by NLRP3 inflammasomes, can cleave IL-1β precursors to form active IL-1β [36]. In our study, the expression levels of phosphorylated NF-κB and IL-1β were reduced to varying degrees in aorta after SAA intervention, suggesting that SAA can reduce the production of IL-1β, a downstream product of NLRP3 inflammasome. We summarize the possible mechanisms of SAA in Figure 8. Consistent with our findings, multiple studies have found that the inhibition of NLRP3 inflammasome by different compounds can significantly impede the development of AS [37,38]. At the same time, experimental studies in different disease models have found that SAA significantly inhibits the activation of NLRP3 inflammasome [15,39]. These results further confirmed the potential value of SAA in treating NLRP3 inflammatory bodies as targets in AS treatment.

In conclusion, our results showed that SAA significantly inhibited early AS in HFD-fed ZDF rats with VD_3_ injections, which may be associated with its ability to control blood lipids and reduce inflammatory responses. AS-associated histopathological changes were also observed in aortic tissues. Furthermore, SAA suppressed the activation of NLRP3 inflammasome and NF-κB signaling in aortic tissues of ZDF rats. Collectively, this study suggested that SAA has potential to alleviate AS and T2DM in ZDF rats.

## Figures and Tables

**Table 1:** Effects of SAA on insulin resistance and atherosclerosis index in T2DM ZDF rats with AS ^1^.

Index	Normal Group	Model Group	ATV Group	SAA Group (0.5 mg/kg)	SAA Group (1 mg/kg)
HOMA-IR	4.01 ± 0.42	13.18 ± 1.39 *	9.43 ± 0.66	11.72 ± 1.16	12.00 ± 0.58
HOMA-IS (×10^−3^)	11.87 ± 1.55	3.49 ± 0.31 *	4.51 ± 0.43	3.55 ± 0.39	3.72 ± 0.19
Atherosclerosis Index	0.71 ± 0.02	6.28 ± 0.32 *	3.91 ± 0.42 ^#^	4.30 ± 0.53 ^#^	4.14 ± 0.61 ^#^

^1^ Data are expressed as mean ± SEM (n = 6). * *p* < 0.05 vs. normal group (Zucker lean control rats). ^#^ *p* < 0.05 vs. model group (Zucker diabetic fatty rats with HFD and VD_3_ injection).

**Table 2:** Effects of SAA on serum hs-CRP (ng/mL) in T2DM ZDF rats with AS ^1.^

Group	Time			
	0 Week	2 Weeks	4 Weeks	6 Weeks
Normal group	84.61 ± 3.18	83.52 ± 3.05	82.40 ± 3.03	84.12 ± 3.76
Model group	118.01 ± 5.66	198.33 ± 4.33 *	207.16 ± 3.86 *	198.15 ± 11.28 *
ATV Group	110.52 ± 6.63	172.58 ± 6.02 ^#^	184.36 ± 5.78 ^#^	176.33 ± 6.80
SAA Group (0.5 mg/kg)	116.58 ± 4.87	193.86 ± 6.06	189.03 ± 5.26 ^#^	151.55 ± 6.79 ^#^
SAA Group (1 mg/kg)	109.37 ± 5.83	199.33 ± 5.90	179.51 ± 3.08 ^#^	152.95 ± 8.53 ^#^

^1^ Data are expressed as mean ± SEM (n = 6). * *p* < 0.05 vs. normal group (Zucker lean control rats). ^#^ *p* < 0.05 vs. model group (Zucker diabetic fatty rats with HFD and VD_3_ injections).
